# A Systematic Review of Oral Modifications Caused by the Prolonged Application of Continuous Positive Airway Pressure (CPAP) and Intraoral Appliances in Patients with Obstructive Sleep Apnea (OSA)

**DOI:** 10.1155/2024/9361528

**Published:** 2024-02-23

**Authors:** Niloofar Jafarimehrabady, Andrea Scribante, Patrizia Defabianis, Giuseppe Merlati, Marina Consuelo Vitale

**Affiliations:** ^1^Department of Clinical, Surgical, Diagnostic and Pediatric Sciences, Section of Dentistry, Unit of Orthodontics and Pediatric Dentistry, University of Pavia, Pavia, Italy; ^2^Department of Surgical Sciences, C.I.R. Dental School-Section of Pediatric Dentistry, University of Turin, Turin, Italy

## Abstract

**Objective:**

Prolonged use of oral devices as a substitute for traditional treatments has been studied in relation to the dental and skeletal changes associated with obstructive sleep apnea syndrome (OSA), which is a sleep-breathing disorder.

**Materials and Methods:**

A review of articles indexed in PubMed, Google Scholar, Cochrane Library, Scopus, Web of Sciences, and CINHAL databases in September 2022 based on MeSH-based keywords with “dental and skeletal” and “oral appliance” and “obstructive sleep apnea” was examined to ensure that the keywords alone or cross-linked, depending on which base of the searched data, were used. 16 articles out of 289 articles were included in the research, and 273 articles were excluded due to lack of study.

**Conclusions:**

CPAP treatment has limited dental or skeletal effects in short-term or long-term use. OAs and MADs show significant dental changes with prolonged use. MAS and TSD are more effective in short-term goals than CPAP. OAs' increase may cause dental and skeletal changes. MPD shows notable cephalometric alterations.

## 1. Introduction

Obstructive sleep apnea (OSA) is a type of sleep disorder that affects breathing. It is characterized by repeated episodes during the night where the upper airway is either partially or completely blocked, causing reduced airflow (hypopnea) or temporary cessation of breathing (apnea). These events result in inadequate ventilation of the alveoli, leading to decreased oxygen levels [1, 2]. In details, OSA is a type of sleep disorder that disrupts breathing due to weakened pharyngeal muscles, leading to reduced or stopped airflow despite persistent attempts to breathe. Most people with OSA experience loud and repetitive snoring, followed by brief pauses in breathing, during which airflow decreases and the upper airway becomes obstructed by a buildup of air in the throat [3].

According to a systematic review, the average prevalence in the general adult population was between 6% and 17%, with an average of 49% in the advanced ages. Obese men and women also had a greater prevalence of OSA. The overall body of evidence proves that the prevalence of OSA increases with age, male sex, and a higher body-mass index [4].

A study in Hong Kong found that embryonic secondary palate was a significant risk factor for OSA [5].

One of the prevalent signs of this condition is the presence of excessive daytime sleepiness (EDS) and fatigue [6]. Other symptoms of OSA include struggling to stay asleep, waking up too early in the morning, and having difficulty falling asleep [7]. The occurrence of this condition is twice as common in men compared to women, and its symptoms tend to worsen with age [3] with a prevalence of up to 60% among individuals aged 60 years and older. Reported data indicates that the prevalence of this condition among men aged 65-69 is 90% [1].

The diagnosis of OSA was addressed by the American Academy of Sleep Medicine (AASM), which proposed two methods in their guidelines. The first method involved using parameters for polysomnography (PSG) symptoms and related methods, which were last updated in 2005. The second method included clinical guidelines for utilizing unattended portable monitors for diagnosing OSA in adult patients. A workgroup of content specialists was assigned by the academy to provide updated clinical practice guidelines (CPG) on this topic. The objectives of the CPG included consolidating and updating information from previous guidelines on OSA diagnosis, such as optimal conditions for conducting laboratory PSG or home sleep apnea tests (HSAT) [8].

In case left untreated, this condition can result in various health complications including cardiovascular diseases, brain diseases, hypertension, metabolic disorders, cognitive disorders, depression, memory loss, and, ultimately, mortality [9]. There are several effective treatments for OSA, which may include weight loss and exercise, nasal obstruction removal, avoiding alcohol, positive airway pressure (PAP) therapy, using oral appliances that reposition the jaw forward, and modifying pharyngeal soft tissue or facial bone to enlarge the upper airway [6].

Early diagnosis of OSA is crucial as it can significantly reduce life expectancy and quality of life. Therefore, therapeutic methods, such as positive airway pressure (PAP) therapy, are commonly employed to effectively manage the condition [10]. The use of this method, which involves nasal administration, is considered the gold standard in OSA management [11]. In order to promote compliance with treatment, moisturizers are employed in this approach. Additionally, treatment adherence may be influenced by other factors such as age, disease severity, and obesity up to a BMI of 35. However, it has been observed that treatment dissatisfaction and discontinuation are more commonly reported among female patients and those with hypertension. Consequently, this trend may contribute to an increase in mortality rates [12].

To mitigate and lower the impact of these factors, patients may be advised to consider alternative methods. One such option is the use of an oral appliance as a treatment alternative. Oral appliances are known to have fewer side effects, higher tolerance levels, and greater patient satisfaction compared to the previously mentioned methods [13]. CPAP masks can be nasal, oro-nasal, or fullface, because mainly nasal and oro-nasal appliances may have effect on the dentition [14].

When evaluating the two options, nasal continuous positive airway pressure (CPAP) is found to have higher adherence and greater reduction in disease severity compared to oral appliances. Warm moisturizing can help alleviate side effects associated with positive airway pressure (PAP). However, oral appliances are preferred due to lower pain levels and, in some cases, dissatisfaction with symptom improvement in the alternative method [12]. Research has indicated that patients tend to favor oral appliances over CPAP or other treatments due to the comfort they provide and the resulting improvement in their quality of life [15, 16].

The mandibular repositioning appliance (MRA) is a widely used oral appliance that is primarily used to advance the mandible and reduce upper airway collapse in patients with obstructive sleep apnea (OSA). There is evidence that this method is effective in treating mild, moderate, and severe cases of sleep apnea. These custom-made appliances are designed to provide precise alignment and function as stabilizers, preventing unwanted movement due to teeth-related problems. It is worth noting that the movement of the tongue may not always synchronize with the mandible while using MRA [17, 18]. Despite having specific goals, these appliances are not a homogeneous group because they vary significantly in design and function. However, in all of these appliances, the tongue is impacted either directly through the advancement of its muscles or indirectly through the advancement of the mandible [19].

These appliances are commonly associated with temporomandibular joint pain, increased salivation, dry mouth, and toothaches. It is common for these side effects to subside with time, however [20].

Apart from reversible and short-term side effects, the use of oral appliances (OAs) may also result in irreversible side effects, such as mandibular protrusion leading to skeletal and dental changes. These changes can be assessed through various imaging techniques and measurements of the mean change [21]. Typically, skeletal changes tend to manifest within 1-3 years, while dental changes may become noticeable after three years [22]. Smith's research, which involved cephalometric analysis, examined also the skeletal, dental, and soft tissue changes resulting from the use of oral appliances (OAs). The findings indicated significant dental changes, including a reduction in overjet, overbite, retroclination of maxillary incisors, and tuberosity of mandibular incisors. However, no clinically significant skeletal changes were reported in relation to the use of OAs [23].

Given the discrepancies in findings from previous studies, this research is aimed at investigating the long-term effects of oral appliances for obstructive sleep apnea (OSA) on dental and skeletal changes.

## 2. Materials and Methods

The search strategy and selection procedures of this systematic review included the dental and skeletal changes that occurred due in the long run of oral appliances for OSA treatment. The search was carried out in June 2022 in English, disregarding the 2010 to 2022 time interval, to study published or in-publish articles. PubMed, Google Scholar, Cochrane Library, Scopus, and Web of Sciences databases were systematically studied. Then, a manual review was conducted regarding other articles and their reference.

**Figure 1:**
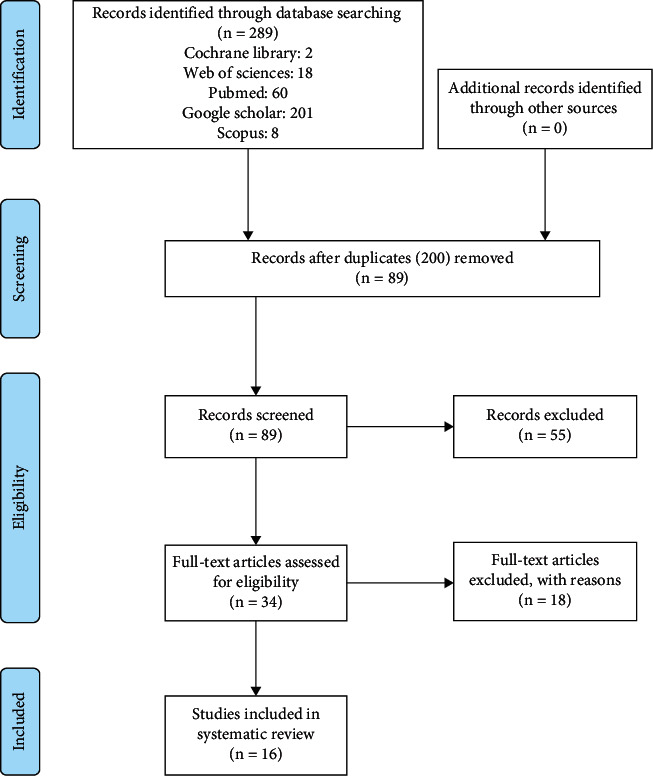
PRISMA flow diagram.

The keywords were selected from the MeSH thesaurus to search the articles. Moreover, free keywords were used to search articles along with the MeSH keywords. The keywords used to search (“dental and skeletal”) and (“oral appliance”) and (“obstrructive sleep opnea”) were examined via + or AND. Afterward, the research results were provided in a PRISMA chart (Figure 1). To make sure regarding the used keywords, they searched again as cross-links, taking into account which database was researched.

Upon confirming the related information, in terms of title and content, a checklist was provided as a table to extract information, and the different properties of trials were recorded in them. To prevent bias, all stages of extraction and examination of references were carried out by two independent researchers. Figure 1 shows the process of investigating and including the articles.

### 2.1. Inclusion Criteria

#### 2.1.1. Types of Studies

A clinical trial with parallel groups enjoying full texts in English was included in the study.

#### 2.1.2. Types of Participants

The various types of participants were as follows: individuals with long-term use of oral appliances for OSA or CPAP.

#### 2.1.3. Types of Interventions

Clinical trial interventions were used to examine oral appliances used to control OSA.

### 2.2. Exclusion Criteria

Studies that used other methods to control OSA.

### 2.3. Quality Assessment and Data Extraction

Two authors examined the credibility and quality of the articles separately. In case of disagreement, they reached a conclusion via discussion and consulting a third author (corresponding author). In order to assess the quality of the articles, the *Cochrane Handbook for Systematic Reviews of Interventions version 5.2.0* was utilized (updated June 2017). The tool is commonly used to assess the quality of clinical trial articles. A key feature of this tool is that it can detect any type of bias, such as selection bias, performance bias, assessment bias, attrition bias, reporting bias, and other forms of bias. Data extracted from the trials includes the study ID (first author's name and the year), study plan, number of participants, inclusion criteria, intervention properties, measured outcomes, ethics approval, and financing. As shown in Table 1, the properties of the included studies are summarized, as is the judgment of the authors regarding the risk of bias in the studies.

### 2.4. Findings

The first stage of the study involved collecting all 289 articles from the databases, among which 233 English cases were studied. Then, 200 were eliminated after checking the title due to being irrelevant and repetitive. The abstracts of articles were studied, and then, 55 other articles were eliminated due to being irrelevant. Then, 18 articles were eliminated since they were not a clinical trial or lack of access to their full texts. Finally, 16 remained and were included in the research.

The research of Minagi et al. investigated the factors predicting dental changes due to the long-term use of OAs in patients with OSA. Night PSG was used to measure the dental changes. For this purpose, 64 people with an average age of 57.7 ± 14.2, who had been treated for OSA for 4.3 ± 2.1 years, participated in this study. Items included in the study are as follows: (1) polysomnography—it was performed as a pretreatment baseline in a sleep laboratory in a sleep center (AHI 0.5 events per hour); (2) the patients that were instructed to use OA during sleep for more than 5 hours a night and more than 5 nights a week; (3) also the patients that were patients who used OA for at least 1 year. In their study, the researchers found that there was a significant reduction in overbite and overjet during the treatment period as well as an increase in the line connecting the lower incisors to the mandible. There was a correlation between this reduction in overjet and the duration, frequency, and progression of mandibular OAs [24].

Doff et al. assessed the probable changes in the face and skull morphology long-term exposure to the adjustable OAs in comparison to CPAP in patients with obstructive sleep apnea/hypopnea. A digital lateral cephalogram with a baseline to determine the cephalometric changes concerning skull and face morphology was performed to measure the probable changes. The OA used in this research was a Thornton adjustable positioner (Airway Management Inc., Dallas, TX, USA) [25].

Hamoda et al. studied the extent and advancement of dental and skeletal changes due to the long-term use. The measurement tools were demographic data and radiographic images. OAM was used by patients in this research [26].

Gong et al. investigated the effectiveness and safety of the long-term use of OAs in the treatment of OSAHS in accordance with the treatment duration. They used PSG, radiography, and questionnaires to examine the mental effects and side effects of OAs [27].

Alessandri-Bonetti et al. assessed the dental-skeletal changes caused by the long-term and consistent use of a mandibular advancement device (MAD) in patients with OSA. This research used a cephalometric measurement tool and a 3D model analysis once at the beginning and then within 1.1 ± 3.5 years, and its correlation with snoring and OSA under treatment by the Silensor device [28].

Tsuda et al. studied the skull changes in adults with OSA after using nCPAP. The measurement tools in this study were basic PSG and a lateral cephalometric radiograph (LCR) [29].

Fransson et al. studied the effect of the mandibular protruding device (MPD) in patients suffering from OSA and snoring. The measurement tool used in this study was one-dimensional basic cephalometric radiography [30].

Venema et al. examined the changes in dental obstruction regarding the long-term use of MAD and CPAP. TAP measurement tools were included (Airway Management Inc., Dallas, Texas, USA). The MAD measurement tools were SomnoDent (Somnomed AG, Australia) [31].

According to Pliska et al., long-term treatment of the mandibular advancement splint (MAS) in patients with OSA causes a number of dental changes. Overbite, overjet, dental arch width, and the relationship between dental arches were measured using a Digital Caliper in this study [32].

Eid and Seif El-Din compared the obstruction changes due to dental side effects between the OA and CPAP that used to treat the OSA. This study used PSG to measure the changes [33].

Ang and Dreyer examined and compared dental changes caused by monoblock and double-block appliances; an Electronic Digital Caliper, with a 0.01 mm scale, was used in this research [34].

Heda was concerned with the periodontal changes due to the treatment of OAM for 4.5 or more years in people with OSA. Research tools were dental records and molds [35].

Kim et al. analyzed and determined the changes in the position of the dental and skeletal structures in the images of cone-beam computed tomography (CBCT). This research used Carestream CS9300 CBCT scans with a voxel size of 0.3 mm [36].

The dental and skeletal effects in patients with OSAHS were investigated also by Laborde et al., after wearing MAD and with regard to the appliance, i.e., hard or semihard. The cephalometric criteria on lateral cephalogram and radiographic photos before and a minimum of 6 months after the treatment were used as the measurement tool [37].

Fransson et al. measured and assessed the position and obstruction of teeth due to 10-year use of MPD at night in patients suffering from OSA or snoring. This study used alginate impressions of the jaw, as well as dental plasters made using an index obtained at an intercuspal position (IP) [38].

Alessandri-Bonetti et al. tried to identify the prevalence of temporomandibular disorders (TMD) in untreated OSA patients. They compared the results to healthy individuals in terms of sex and age [39].

### 2.5. Methodological Quality

Among the total articles studied in this research, eight of them were retrospective [24, 26–28, 32, 35–37]. There were five prospective studies [29, 30, 34, 38, 39], and the three remaining were controlled random studies [25, 31, 33]. Except for one article, the rest did not report any attrition [30].

Several articles did not include reports on ethics and funding [26, 33, 34, 37].

## 3. Results

A total of 1258 case studies were studied, and the results are outlined in Table 1. For patients with OSA, MAS and TSD treatments seem to be more qualified than CPAP treatment in short treatment. Long-term use of an oral appliance resulted in small but significant (dental) changes rather than CPAP.

A significant decrease and change were observed in overbite and overjet, as the results of MASs (included 77 patients) and MPDs (included 77 patients).

In oral appliance and CPAP treatment, no changes in skeletal variables were found, while in prolonged OAM use and MAD treatment, negligible and insignificant skeletal changes were found.

Overall, the studies suggested that long-term use of OAs, CPAP, and MADs can result in remarkable dental changes, while skeletal changes may vary and may not be significant in some cases. Treatment duration and patient characteristics were identified as potential predictors of the observed changes.

## 4. Discussion

The present review found that CPAP treatment did not yield considerable dental or skeletal changes, as reported in studies involving both short-term and long-term use of CPAP. In contrast, long-term uses of OAs and MADs were associated with remarkable dental changes, suggesting that prolonged use of these treatments (without considering the age of participants) may be necessary to achieve optimal results in terms of dental changes. The effectiveness of treatments for OSA has been a subject of extensive research, with various modalities being explored. In particular, MAS, TSD, and CPAP have emerged as popular and efficient options for managing OSA. Patient characteristics, such as age, severity of OSA, and underlying skeletal structure, were also found to potentially influence the effectiveness of the treatments [40]. Long-term OA therapy may cause dental movement and skeletal changes, leading to a reduction in overjet and overbite. These mechanical side effects are caused by the reciprocal forces applied to the teeth and jaw by the OA device. In the future, patients may experience aesthetic concerns or difficulties with chewing and biting due to these changes [41]. One significant finding from the research is that MAS and TSD treatments appear to be more effective than CPAP in achieving short-term treatment goals [42, 43]. Although CPAP can effectively reduce the severity of OSA, a higher number of patients are choosing OA, as it has been shown to yield superior results in severe cases, particularly with adjustable OA [44]. Treatment with OAs in OSA may lead to a significant increase of the upper airway volume with a subsequent decrease of AHI [43]. MAS and TSD treatments have been shown to result in small but meaningful dental changes, as evidenced by observed decreases and changes in overbite and overjet. MAS and TSD treatments showed small but substantial dental changes compared to CPAP. However, in the MAS group, overbite and overjet were considerably reduced [44]. For the OA and CPAP, despite their similarities in clinical practice, OA may not be able to completely eliminate all obstructive events during sleep, unlike CPAP [45]. Individuals with MPD demonstrated notable alterations in all cephalometric measurements, with the exception of maxillary protrusion. Significant changes, including reduced overjet and overbite, were observed in the MPD group [30]. We had some limitations. The patient characteristics such as age, severity of OSA, and underlying skeletal structure may influence treatment outcomes. Also, we did not find out the exact time period in all articles because some participants passed away before finishing the period of scheduled treatment. Controlling for these variables may be challenging. Dental and skeletal changes can be difficult to measure objectively, particularly over a long period of time. Considering measurement techniques needs reliable and valid documentation, particularly on long-term data included in the analysis. Mandibular advancement devices (MAD), tongue repositioning devices, rapid maxillary expansion (RME), and multiple other appliances are cases of OA treatment. Much further investigation on MAD is necessary since this category type of technology, MAD, is already broad and may include numerous such as twin-block appliances and Herbst appliances. On this topic, it would be wise to keep adults and children asunder so that more reviews are required.

To draw firm conclusions and examine how patient skeletal structure may influence treatment outcomes, additional studies evaluating gnathological parameters of patients with apnea using an advancement device and well-designed studies are required. Additionally, it should be considered that OSAS can promote enamel demineralization and periodontal problems [2]. Therefore, future research perspectives could include the evaluation of fluoride [46], casein phosphopeptide-amorphous calcium phosphate [47], biomimetic hydroxyapatite [48], and other remineralizing agents in patients with OSAS. Finally, also the use of lysates [49], probiotics [50], or other natural compounds [51] that can have an influence on oral microbiota has to be tested in patients with OSAS in order to help in the maintenance of periodontal health.

## 5. Conclusion

CPAP treatment has limited dental or skeletal effects in short-term or long-term use. OAs and MADs show significant dental changes with prolonged use. MAS and TSD are more effective in short-term goals than CPAP. Long-term utilization of OAs, CPAP, and MADs can result in exceptional dental changes, whereas skeletal changes may change and may not be noteworthy in a few cases. In MAS treatment users, overbite and overjet were significantly decreased.

## Figures and Tables

**Table 1:** Properties of the included studies.

Minagi et al. [24]		
Methods	Study design: retrospective study Trial location: Japan	
Participants	Number of participants: 64 patients Eligibility criteria: average age at start of treatment: 57.7 ± 14.2 years. The average duration of treatment was 4.3 ± 2.1 years	
Intervention	Intervention: participants with OSA who received long-term oral treatment	
Outcome	Primary outcome: As a result of the complete treatment, there was a significant reduction in overjet (1.5 ± 1.3 mm) and overbite (0.90 ± 1.5 mm), and an increase in the lower incisor line to the mandibular plane (3.1 ±5.4°). Treatment duration, use frequency, and mandibular advancement of the OAs were associated with greater reductions in OJ by 1 mm Secondary outcome: no significant skeletal changes were found after long-term treatment	
Notes	Ethics approval: Osaka University Dental Hospital, No. H28-E20	

Risk of bias		
Bias	Authors' judgment	Support for judgment
Random sequence generation (selection bias)	High risk	Allocation was not random
Allocation concealment (selection bias)	High risk	Subjects were paired according to age, type of surgery, educational attainment, and operative experience
Blinding of participants and personnel (performance bias)	High risk	There was no possibility of blinding
Blinding of outcome assessor (detection bias)	Low risk	Blinding is done
Incomplete outcome data (attrition bias)	Low risk	Due to the retrospective nature of the study, it was not possible to exclude participants from the study.
Selective reporting (reporting bias)	Low risk	All the prespecified outcomes in the method section were addressed adequately
Other biases		Work for this study was performed at the Osaka University Graduate School of Dentistry. The authors report no conflicts of interest.

Doff et al. [25]		
Methods	Study design: randomized controlled study Trial location: Netherlands	
Participants	Number of participants: 103 participants, age: 49 ± 10 years Eligibility criteria: Participants were individuals with obstructive sleep apnea syndrome who had received oral therapy or positive airway pressure therapy (CPAP).	
Intervention	Intervention: Fifty-one participants were randomized to oral appliance therapy Control: 52 participants to CPAP therapy Treatment was at least 2 years	
Outcome	Primary outcome: An oral appliance, in comparison with CPAP, resulted in small but significant (dental) changes. The lower and total anterior facial height increased significantly, by 0.8 (±1.5) mm and 0.9 (±1.4) mm, respectively The secondary outcome was the absence of any changes in skeletal variables.	
Notes	Ethics approval: the Groningen University Medical Center's Ethics Committee	

Risk of bias		
Bias	Authors' judgment	Support for judgment
Random sequence generation (selection bias)	Low risk	Allocation was random
Allocation concealment (selection bias)	High risk	Participants were divided into two groups: oral appliance therapy and CPAP therapy.
Blinding of participants and personnel (performance bias)	High risk	Due to the nature of the study, it was not possible to blind
Blinding of outcome assessor (detection bias)	Unclear	Blinding is not explained.
Incomplete outcome data (attrition bias)	High risk	31 and 37 patients were divided into oral group and CPAP group, respectively. Other subjects were excluded from the study
Selective reporting (reporting bias)	Low risk	In the method section, all of the prespecified outcomes were adequately addressed
Other biases		Ethics approval for the present study was obtained from the Ethics Committee of Groningen University Medical Center. Each patient provided written informed consent prior to enrolment No information has been provided regarding the funding organization

Hamoda et al. [26]		
Methods	Study design: retrospectively study Trial location: Columbia	
Participants	Number of participants: 62 participants, average age at start of treatment: 49 ± 8.6 years Eligibility criteria: adults with mild to severe wheezing or OSA. At least 8 years have passed since treatment	
Intervention	Subjects had been receiving OAM therapy for at least 8 years	
Outcome	Primary outcome: that there are significant and progressive dental changes with prolonged OAM use. Over the same time period, skeletal or postural changes were negligible. Secondary outcome: treatment duration was the predictor consistently associated with the magnitude of the observed side effects	
Notes	Ethics approval	

Risk of bias		
Bias	Authors' judgment	Support for judgment
Random sequence generation (selection bias)	High risk	Allocation was not random
Allocation concealment (selection bias)	High risk	Due to the nature of the study, it was not possible to conceal the allocation
Blinding of participants and personnel (performance bias)	High risk	There was no possibility of blinding
Blinding of outcome assessor (detection bias)	Unclear	Blinding is not explained.
Incomplete outcome data (attrition bias)	Low risk	All the prespecified outcomes in the method section were addressed adequately
Selective reporting (reporting bias)		All the prespecified outcomes in the method section were addressed adequately
Other biases		No funding was provided for this study Ethics approval has been obtained. There was no conflict of interest.

Gong et al. [27]		
Methods	Study design: retrospective study Trial location: China	
Participants	Number of participants: 412 participants. Eligibility criteria: participants with OSAHS receiving OA treatment	
Intervention	412 participants with OSAHS receiving OA treatment	
Outcome	Primary outcome: Following the OA, the apnea-hypopnea index values were reduced from a median of 24.50 (quartiles, 14.65, 54.05) to 7.40 (2.12, 10.00) at baseline and to 4.25 at follow-up, respectively. Secondary outcome: Cephalometric analysis indicated mild and slow changes in the skeleton and occlusion after 5 years.	
Notes	Ethics approval: UBC Clinical Research Ethics Board H11-01661.	

Risk of bias		
Bias	Authors' judgment	Support for judgment
Random sequence generation (selection bias)	High risk	Allocation was not random
Allocation concealment (selection bias)	High risk	Subjects were paired according to age, type of surgery, educational attainment, and operative experience
Blinding of participants and personnel (performance bias)	High risk	There was no possibility of blinding
Blinding of outcome assessor (detection bias)	Unclear	Blinding is not explained.
Incomplete outcome data (attrition bias)	Low risk	All the prespecified outcomes in the method section were addressed adequately
Selective reporting (reporting bias)	Low risk	All the prespecified outcomes in the method section were addressed adequately
Other biases		The research was partly supported by the Capital Characteristics of Clinical Application Fund (D101100050010019). Ethics approval has been obtained.

Alessandri-Bonetti et al. [28]		
Methods	Study design: retrospectively study Trial location: Italy	
Participants	Number of participants: 20 participants Eligibility criteria: MAD therapy for more than 2 years without treatment discontinuation during the whole study period, MAD use for a minimum of 5 nights per week and usually for the entire night (assessed through a questionnaire administered at the long-term follow-up day), and availability of good quality study models and lateral cephalograms at the baseline and follow-up visits	
Intervention	Participants had mild, moderate, and severe snoring that lasted an average of 3.5 ± 1.1 years.	
Outcome	Primary outcome: the maxilla revealed a significant decrease in horizontal position and a significant retroclination of the upper incisor, while the mandible displayed a significant downward rotation and a proclination of the lower incisor Secondary outcome: decrease in upper total space discrepancy, overjet, and overbite. In the regression analysis, treatment time influenced the lower incisor inclination and OJ; participants' initial characteristics had an effect on OJ.	
Notes	Ethics approval: This study was approved by the Institutional Review Board (N. 268/15)	

Risk of bias		
Bias	Authors' judgment	Support for judgment
Random sequence generation (selection bias)	High risk	Allocation was not random
Allocation concealment (selection bias)	High risk	Due to the nature of the study, it was not possible to conceal the allocation
Blinding of participants and personnel (performance bias)	High risk	There was no possibility of blinding
Blinding of outcome assessor (detection bias)	Unclear	Blinding is not explained.
Incomplete outcome data (attrition bias)	Low risk	All the prespecified outcomes in the method section were addressed adequately
Selective reporting (reporting bias)	High risk	Supplementary material is available at the European Journal of Orthodontics online.
Other biases		No funding was provided for this study. There was no conflict of interest

Tsuda et al. [29]		
Methods	Study design: prospective study Trial location: Japan	
Participants	Number of participants: 46 participants Eligibility criteria: (1) stop using nCPAP, (2) poor compliance (4 h/d or, 5 d/wk), (3) use of a nasal pillow or fullface type mask, (4) poor quality baseline radiograph for evaluation.	
Intervention	Both baseline and follow-up cephalometry radiographs were obtained from 46 Japanese subjects who used nCPAP for at least two years. These two radiographs were analyzed, and changes in the craniofacial structures were assessed.	
Outcome	Primary result: Cephalometric variables after nCPAP use showed significant anterior maxilla retrusion, decreased maxillary to mandibular discrepancy, supramental and chin position setback, maxillary incisor retroclination, and decreased facial convexity. Secondary result: No significant correlations were observed between craniofacial alterations, demographic characteristics, or nCPAP duration and no self-reported permanent changes in occlusion and facial profile	
Notes	Ethics approval: The study protocol was approved by the local Kirigaoka Tsuda Hospital review board	

Risk of bias		
Bias	Authors' judgment	Support for judgment
Random sequence generation (selection bias)	Unclear	How to randomize participants is not mentioned.
Allocation concealment (selection bias)	High risk	Due to the nature of the study, it was not possible to conceal the allocation
Blinding of participants and personnel (performance bias)	High risk	There was no possibility of blinding
Blinding of outcome assessor (detection bias)	Unclear	Blinding is not explained.
Incomplete outcome data (attrition bias)	Low risk	All prespecified results under the method section were satisfactorily addressed
Selective reporting (reporting bias)	Unclear	All prespecified results in the method section were satisfactorily addressed.
Other biases		Funding/support: Financial support for this study was received from the MITACS Graduate Research Internship Program. A MITACS Accelerate Internship Grant supported in part the postdoctoral fellow salary of H. Tsuda. No funding was provided for this study.

Fransson et al. [30]		
Methods	Study design: prospective study Trial location: Sweden	
Participants	Number of participants: 77 people were included in the study Eligibility criteria: sufficient number of teeth to retain an MPD, good dental health, and a maximum protrusion range of $6 mm as measured with the George Gauge instrument (Boos Dental Laboratories, MN).	
Intervention	Use of the mandibular protrusion device (MPD) in people with obstructive sleep apnea	
Outcome	Primary outcome: The degree of deviation of the maxillary incisors decreased Secondary outcome: Significant changes in overjet and overbite reduction were observed in the MPD group	
Notes	Ethics approval: The study is endorsed by the Orebro University Ethics Committee	

Risk of bias		
Bias	Authors' judgment	Support for judgment
Random sequence generation (selection bias)	High risk	The distribution was not randomly chosen.
Allocation concealment (selection bias)	High risk	Experiments were matched based on MPD usage and discontinuation of MPD usage
Blinding of participants and personnel (performance bias)	High risk	After ten years, 12 participants went blind
Blinding of outcome assessor (detection bias)	Unclear	Blinding is not explained
Incomplete outcome data (attrition bias)	Low risk	The method section addressed all the prespecified outcomes.
Selective reporting (reporting bias)	Low risk	The method section addressed all the prespecified outcomes.
Other biases		All subjects provided informed consent after practicing about the study's purpose. The study has indeed been authorised by the Orebro University Ethics Committee. The funding organization has not been disclosed.

Venema et al. [31]		
Methods	Study design: randomized controlled trial Trial location: Netherlands	
Participants	Number of participants: 94 people Eligibility criteria: Participants were individuals with obstructive sleep apnea syndrome who had received oral TAP or SomnoDent or positive airway pressure (CPAP) therapy	
Intervention	Intervention group: 29 people in TAP group, 31 people in SomnoDent and control group, and 34 people in the CPAP group	
Outcome	Primary outcome: The number of occlusal contact points in the premolar region decreased in all three groups Secondary outcome: Overbite changed in all three groups	
Notes	Ethics approval	

Risk of bias		
Bias	Authors' judgment	Support for judgment
Random sequence generation (selection bias)	Low risk	Allocation was random
Allocation concealment (selection bias)	High risk	Due to the nature of the study, it was not possible to conceal the allocation
Blinding of participants and personnel (performance bias)	High risk	Due to the nature of the study, it was not possible to blind
Blinding of outcome assessor (detection bias)	Unclear	Blinding is not explained.
Incomplete outcome data (attrition bias)	High risk	29 participants in TAP group, 31 participants in SomnoDent, and 34 participants in CPAP group were excluded from the study
Selective reporting (reporting bias)	Low risk	All outcomes clarified in the method section were fully explained.
Other biases		The funding organization has not been reported The authors report no conflicts of interest

Pliska et al. [32]		
Methods	Study design: retrospective study Trial location: Canada	
Participants	Number of participants: 77 people with a mean age 47.5 ± 10.2 Eligibility criteria: has been treated with OSA for at least 8 years using MAS, continuous use of the device at night	
Intervention	Treatment of obstructive sleep apnea (OSA) using long-term treatment of mandibular progressive splint (MAS)	
Outcome	Primary outcome: Over time, a significant decrease was observed in overbite and overjet Secondary outcome: The overjet decreased continuously with a constant trend over time	
Notes	Ethics approval: The Clinical Research Ethics Board of the University of British Columbia approved this study.	

Risk of bias		
Bias	Authors' judgment	Support for judgment
Random sequence generation (selection bias)	High risk	Allocation was not random
Allocation concealment (selection bias)	High risk	Subjects were paired based on the history of MAS use and the presence of dental plaster
Blinding of participants and personnel (performance bias)	High risk	There was no possibility of blinding
Blinding of outcome assessor (detection bias)	Unclear	Blinding is not explained
Incomplete outcome data (attrition bias)	Low risk	Due to the retrospective nature of the study, it was not possible to exclude participants from the study
Selective reporting (reporting bias)	Low risk	All reports are given in the measurement method section
Other biases		It was not an industry-supported study. The University of British Columbia garnered assistance in the form of oral appliance royalties and a UBC Undergraduate Student Summer Internship Award. The authors have reported no financial conflicts of interest. The research was performed out at the Department of Oral Health Sciences, Faculty of Dentistry, University of British Columbia, Vancouver, BC, Canada.

Eid et al. [33]		
Methods	Study design: a randomized trial study Trial location: Egypt	
Participants	Participants: 31 people over 20 years old Eligibility criteria: having OSA, use alternative therapy at any time during follow-up, using oral devices for more than 5 nights a week and more than 6 hours a night	
Intervention	10 participants in the maxillary splint (MAS) group, 10 participants in the tongue stabilization device (TSD), and 11 participants in the control group for continuous positive airway pressure therapy (CPAP)	
Outcome	Primary outcome: MAS and TSD treatment showed small but significant dental changes compared to CPAP. Secondary outcome: In the MAS group, overbite and overjet were significantly reduced	
Notes	Ethics approval: not reported	

Risk of bias		
Bias	Authors' judgment	Support for judgment
Random sequence generation (selection bias)	Low risk	The distribution was random.
Allocation concealment (selection bias)	High risk	Due to the nature of the study, it was not possible to conceal the allocation
Blinding of participants and personnel (performance bias)	High risk	Due to the nature of the study, it was not possible to blind
Blinding of outcome assessor (detection bias)	Unclear	Blinding is not explained
Incomplete outcome data (attrition bias)	High risk	10 participants in the mandibular progression splint group (MAS), 10 participants in the tongue stabilization device (TSD), and 11 participants in the control group under continuous positive airway pressure (CPAP) were excluded from the study
Selective reporting (reporting bias)	Low risk	All the prespecified outcomes in the method section were addressed adequately
Other biases		Written informed consent was obtained from each patient before enrolment The funding organization has not been reported

Ang et al. [34]		
Methods	Study design: prospective study Trial location: Australia	
Participants	Number of participants: 52 Eligibility criteria: subjects with maxillary and mandibular first permanent molars and canines. Subjects who had worn a MAS continuously (minimum of five to six hours per night) for at least 6 months	
Intervention	17 subjects wore soft elastomeric monoblock appliances, and 29 subjects wore hard acrylic	
Outcome	Primary outcome: measurement of dental and arch changes on study models using standard biting radiographs Secondary outcome: BD users decreased in the distance between maxillary canines and increased in the distance between mandibular molars	
Notes	Ethics approval: not reported	

Risk of bias		
Bias	Authors' judgment	Support for judgment
Random sequence generation (selection bias)	High risk	Allocation was not random
Allocation concealment (selection bias)	High risk	Subjects were paired based on the use of monoblock and double-block devices
Blinding of participants and personnel (performance bias)	High risk	There was no possibility of blinding
Blinding of outcome assessor (detection bias)	Unclear	Blinding is not explained
Incomplete outcome data (attrition bias)	Low risk	All the prespecified outcomes in the method section were addressed adequately
Selective reporting (reporting bias)	Low risk	All the prespecified outcomes in the method section were addressed adequately
Other biases		No funding was provided for this study There was no conflict of interest

Heda et al. [35]		
Methods	Study design: retrospective study Trial location: Canada	
Participants	Number of participants: 21 participants (15 males, mean age 57.4 ± 12.0 y.o), with a mean treatment length of 7.6 ± 3.3 years (range = 4.5 to 14.3 years) Eligibility criteria: participants who wish to participate and have completed their informed consent. Participants who use OAM regularly and for at least 4.5 years. Use OAM for at least 5 nights a week and at least 4 hours a night. Availability of good quality pretreatment records for existing participants. The patient is at least 19 years old or older. The patient is able to understand and communicate in English	
Intervention	Clinical follow-up of teeth through cephalograms and dental molds	
Outcome	Primary outcome: PSR Absence of active periodontal disease using data Secondary outcome: with continuous use of OAM at different evaluation time periods, clinical crown height did not change	
Notes	Ethics approval: University of British Columbia, Vancouver, No. H14-00743	

Risk of bias		
Bias	Authors' judgment	Support for judgment
Random sequence generation (selection bias)	High risk	Allocation was not random
Allocation concealment (selection bias)	High risk	The subjects were paired based on cephalometric information
Blinding of participants and personnel (performance bias)	High risk	There was no possibility of blinding
Blinding of outcome assessor (detection bias)	Unclear	Blinding is not explained
Incomplete outcome data (attrition bias)	Low risk	Due to the retrospective nature of the study, it was not possible to exclude participants from the study
Selective reporting (reporting bias)	Low risk	All reports are given in the measurement method section
Other biases		The study was conducted at UBC Sleep Clinic and a clinic-affiliated private practice The study has a code of ethics

Kim et al. [36]		
Methods	Study design: retrospective study Trial location: Spain	
Participants	Number of participants: 18 people in the age range of 29 to 63 years, including a woman and 17 men Eligibility criteria: use of MAD as a treatment for OSA and availability of images required for this study	
Intervention	CBCT scans of 0.3 mm voxel size were taken with the Carestream CS 9300 Select (Rochester, New York, USA), exposition 80 Kv 4 mA 8.01 s, dose 448 mGy cm2, size 18 *μ*m × 18 *μ*m × 18 *μ*m, and image 10 cm × 10 cm × 10 cm according to manufacturer's settings.	
Outcome	Primary outcome: Symptoms can be seen using CBCT images Secondary outcome: MAD causes skeletal changes	
Notes	Ethics approval: not reported	

Risk of bias		
Bias	Authors' judgment	Support for judgment
Random sequence generation (selection bias)	High risk	Allocation was not random
Allocation concealment (selection bias)	High risk	Subjects were paired based on cone-beam computed tomography (CBCT) images in MAD consumers
Blinding of participants and personnel (performance bias)	High risk	There was no possibility of blinding
Blinding of outcome assessor (detection bias)	Unclear	Blinding is not explained
Incomplete outcome data (attrition bias)	Low risk	Due to the retrospective nature of the study, it was not possible to exclude participants from the study
Selective reporting (reporting bias)	Low risk	All reports are given in the measurement method section
Other biases		This study was performed at the orthodontic clinic of the University of Alfonso X in Madrid No funding was provided for this study

Laborde et al. [37]		
Methods	Study design: retrospective study Trial location: France	
Participants	Number of participants: 22 participants, mean age was 53.3 ± 12.4 years Eligibility criteria: participants had used a rigid or semirigid device for more than six months. The medical record contained a film around the skull before the device was inserted, which is a document showing the initial AHI.	
Intervention	9 people in the semirigid MAD group and 13 people in the rigid MAD group	
Outcome	Primary outcome: reduce overbite and overjet with semirigid MAD Secondary outcome: showed statistically significant differences in dental analysis but insignificant differences in skeletal analysis.	
Notes	Ethics approval: not reported	

Risk of bias		
Bias	Authors' judgment	Support for judgment
Random sequence generation (selection bias)	High risk	Allocation was not random
Allocation concealment (selection bias)	High risk	Subjects were paired according to the type of MAD device
Blinding of participants and personnel (performance bias)	High risk	There was no possibility of blinding
Blinding of outcome assessor (detection bias)	Unclear	Blinding is not explained
Incomplete outcome data (attrition bias)	Low risk	Due to the retrospective nature of the study, it was not possible to exclude participants from the study
Selective reporting (reporting bias)	Low risk	All the prespecified outcomes in the method section were addressed adequately
Other biases		This study was performed in the Department of Oral and Maxillofacial Surgery at the Teaching Hospital of the University of Lille, France. The authors declare that they have no competing interest.

Fransson et al. [38]		
Methods	Study design: prospectively study Trial location: Sweden	
Participants	Number of participants: 77 participants Eligibility criteria: having OSA problems and snoring, sufficient teeth for maintaining MPD, good dental health, and maximum ability to exhaust at least 6 mm using George Gauge®	
Intervention	41 users of PMD, 19 people discontinued PMD	
Outcome	Primary outcome: reduce overjet in both groups Secondary outcome: reduction of overjet and overbite in PMD discontinuation group	
Notes	Ethics approval: The baseline study was approved by the Medical Ethics Committee at Örebro Medical Centre Hospital, Örebro, Sweden.	

Risk of bias		
Bias	Authors' judgment	Support for judgment
Random sequence generation (selection bias)	High risk	Allocation was not random
Allocation concealment (selection bias)	High risk	Use of MPD handles in participants with OSA
Blinding of participants and personnel (performance bias)	Low risk	17 people were excluded from the study after 10 years of follow-up
Blinding of outcome assessor (detection bias)	Low risk	Blinding is not explained
Incomplete outcome data (attrition bias)	Low risk	The method section's predetermined outcomes were all appropriately addressed.
Selective reporting (reporting bias)	Low risk	The method section's predetermined outcomes were all appropriately addressed.
Other biases		No funds provided for this study. Verification of ethics has been obtained, but the ethics code has not been taken. There was no balance of interest. Informed consent form was signed at the 10-year follow-up, following approval by the Regional Ethics Review Board in Uppsala, Sweden.

Alessandri-Bonetti et al. [39]		
Methods	Study design: prospectively study Trial location: Italy	
Participants	Participants: 82 participants Eligibility criteria: age over 18 years of age, having apnea-hypopnea index (AHI) higher than 5	
Intervention	41 people in the OSA group and 41 people in the control group	
Outcome	Primary outcome: The groups differ in TMD, pain, and joint disorder Secondary outcome: No association was found between temporomandibular (TMD) disorders and OSA	
Notes	Ethics approval: Gemelli Hospital approved the protocol with number 7372/18 before starting the trial	

Risk of bias		
Bias	Authors' judgment	Support for judgment
Random sequence generation (selection bias)	High risk	The distribution was not arbitrary.
Allocation concealment (selection bias)	High risk	Subjects were matched in one group based on age, sex, and hospitalization in the ENT department or referring to the oral clinic.
Blinding of participants and personnel (performance bias)	High risk	There was no possibility of blinding
Blinding of outcome assessor (detection bias)	Low risk	Blinding is not explained
Incomplete outcome data (attrition bias)	Low risk	The method section's predetermined outcomes were all suitably addressed.
Selective reporting (reporting bias)	Low risk	The method section's predetermined outcomes were all suitably addressed.
Other biases		There has been no funding given for this investigation. The ethics board has given approval. A conflict of interest will not matter.

## Data Availability

The authors confirm that the data supporting the findings of this study are available within the article.
